# Chronic fertilization of 37 years alters the phylogenetic structure of soil arbuscular mycorrhizal fungi in Chinese Mollisols

**DOI:** 10.1186/s13568-018-0587-2

**Published:** 2018-04-17

**Authors:** Mingchao Ma, Marc Ongena, Qingfeng Wang, Dawei Guan, Fengming Cao, Xin Jiang, Jun Li

**Affiliations:** 1 Institute of Agricultural Resources and Regional Planning, Chinese Academy of Agricultural Sciences, Beijing, 100081 China; 2 Microbial Processes and Interactions Research Unit, Gembloux Agro-Bio Tech, University of Liège, Passage des Déportés, 2, Gembloux, Belgium; 3 Laboratory of Quality & Safety Risk Assessment for Microbial Products, Ministry of Agriculture, Beijing, 100081 China

**Keywords:** Black soil, Inorganic fertilizer, Manure, AMF, Illumina MiSeq sequencing

## Abstract

Arbuscular mycorrhizal fungi (AMF) play vital roles in sustaining soil productivity and plant communities. However, adaption and differentiation of AMF in response to commonly used fertilization remain poorly understood. In this study, we showed that the AMF community composition was primarily driven by soil physiochemical changes associated with chronic inorganic and organic fertilization of 37 years in Mollisols. High-throughput sequencing indicated that inorganic fertilizer negatively affected AMF diversity and richness, implying a reduction of mutualism in plant–AMF symbiosis; however, a reverse trend was observed for the application of inorganic fertilizer combined with manure. With regards to AMF community composition, order *Glomerales* was dominant, but varied significantly among different fertilization treatments. All fertilization treatments decreased family *Glomeraceae* and genus *Funneliformis*, while *Rhizophagus* abundance increased. Plant-growth-promoting-microorganisms of family *Claroideoglomeraceae* and genus *Claroideoglomus* were stimulated by manure application, and likely benefited pathogen suppression and phosphorus (P) acquisition. Family *Gigasporaceae* and genus *Gigaspora* were negatively correlated with available P in soil. Additionally, redundancy analysis further suggested that soil available P, organic matter and pH were the most important factors in shaping AMF community composition. These results provide strong evidence for niche differentiation of phylogenetically distinct AMF populations under different fertilization regimes. Manure likely contributes to restoration and maintenance of plant–AMF symbiosis, and the balanced fertilization would favor the growth of beneficial AMF communities as one optimized management in support of sustainable agriculture in Mollisols.

## Introduction

Arbuscular mycorrhizal fungi (AMF) are ubiquitous in terrestrial ecosystems. They are obligate plant root symbionts found in more than 80% of plant families (Mueller and Bohannan 2015) and they provide multiple benefits for both plant and AMF. For instance, AMF can provide plants with critical nutrients (Hodge et al. 2010) and non-nutrient benefits (Walder and van der Heijden 2015), help plants withstand drought (Li et al. 2013), develop soil aggregates (Rillig and Mummey 2006), stimulate photosynthesis (Kaschuk et al. 2009) and increase plant productivity (Maček et al. 2011), thereby influencing ecosystem processes and agricultural sustainability (Rillig 2004). Moreover, AMF confer resistance to plants against many soil-borne pathogens and various nematodes by their colonization of the plant root (Harrier and Watson 2004; Sikes et al. 2009). The plant, in turn, provides photosynthetic carbon (C) as an energy source for AMF, which can alter the fungal community (Kim et al. 2015). In addition to beneficial effects on plants, AMF also enhance ecosystem sustainability by influencing numerous soil properties and structure (Wilson et al. 2009), including soil stability (Mueller and Bohannan 2015), C storage (Treseder and Allen 2000), soil moisture (Johnson et al. 2003), and nitrogen (N), C and phosphorus (P) cycles (Van Der Heijden et al. 2008). Lower AMF biodiversity can also lead to unsustainable crop production and ecosystem instability (Maček et al. 2011). More importantly, the beneficial effects of AMF decrease under conditions of high fertility (Collins and Foster 2009). Although they are ubiquitous and of ecological importance, AMF have been poorly studied in the important black soils of China.

Black soils, referred to as Mollisols, are one of the most crucial soil resources for crop production (Liu et al. 2012a), due to their key roles in the supply of national staple food (Han et al. 2013). However, excessive cultivation and intensive fertilization have caused serious soil degeneration and substantial losses of soil productivity since the middle of the last century (Singh et al. 2014; Liu et al. 2015a). Intensive inorganic input leads to a shift from fungal-based to more bacterial-based soil food webs (Thiele-Bruhn et al. 2012). Moreover, repeated application of inorganic fertilizer decreases soil pH (Liu et al. 2012b), which in turn can reduce nutrient availability and microbial biomass (Bardgett 2005). Application of organic manure can reduce use of inorganic fertilizers and benefit soil quality by alleviating soil acidification, accumulating soil organic matter (OM), improving soil microbial community structure and reinforcing the self-regulating status of soil systems (Hammesfahr et al. 2011; Insam et al. 2015). The responses of soil bacterial and fungal communities to inorganic fertilizer and manure in black soils were well reported in previous studies (Zhou et al. 2015, 2016; Ding et al. 2016, 2017), but we know less about the effects on AMF, which work as good indicators of the soil ecosystem and affect the soil nutrition cycle and plant health. With chronic fertilizer input, the changes in soil properties no doubt influence AMF microbial communities and shift the plant–AMF symbiosis towards reduced mutualism or parasitism along a continuum (Verbruggen and Kiers 2010), as they could reduce the benefits provided by these symbionts. Understanding the shift of AMF community composition is critical to assessing the impact of fertilization for determining a more effective strategy in sustainable soil fertility. As previously documented, several mycorrhizal fungi are closely associated with the soil P cycle (Smith et al. 2011), and provide the plant hosts with P in the typically available form of phosphate. Given the relationship between AMF and soil available P (AP), and that soil AP, OM and pH are important contributors to the fungal community (Ding et al. 2017), they may also shape the AMF community. In this study, we investigated the chronic (37-year) effects of fertilization regimes on soil properties and the AMF community, and determined the primary factors driving the shifts in the AMF community. We hypothesized the following: (1) chronic inorganic fertilization would significantly decrease AMF diversity; (2) the shifts in AMF community composition would result from fertilizer-driven changes in soil properties, such as AP, pH and OM; and (3) the application of manure would shift the AMF community towards a better structure and diversity, with an opposite effect for inorganic fertilizer.

## Materials and methods

### Field experiment and soil collection

Long-term experiments can provide realistic and effective conditions for obtaining the exact information required to maintain soil quality by determining changes in soil properties and processes (Li et al. 2008). Our field experiment was conducted at the Scientific Observation Station of Arable Land Conservation and Agriculture Environment, Harbin City, Heilongjiang Province, China. Wheat, soybean and maize had been continuously grown on the field since 1980. The soil at the site was classified a clay loam and the experiment had a completely randomized block design with three replications. Blocks were randomized into plots of 9 × 4 m. More information was provided in our previous studies (Zhou et al. 2017). We analyzed soil samples from four treatments: CK (no fertilizer), M (manure), NPK (inorganic N, P and potassium (K) fertilizers) and MNPK (inorganic N, P and K fertilizers plus manure). Inorganic N, P and K fertilizers were applied as urea (75 kg hm^−2^ year^−1^), calcium superphosphate plus ammonium hydrogen phosphate (150 kg hm^−2^ year^−1^) and potassium sulfate (75 kg hm^−2^ year^−1^), respectively. The manure was horse manure applied at 18,600 kg hm^−2^ year^−1^.

In June 2016, after 37 years of fertilization application, we collected the rhizosphere soils during florescence of wheat, when the rhizosphere effects were considered to be most pronounced (Cheng et al. 2003). Twenty wheat plants and their roots were collected from the middle of each replication and shaken gently. The remaining adhering soil was carefully collected and mixed thoroughly as a single rhizosphere sample. Thus, a total of 12 soil samples were obtained for further analyses. Each soil sample was passed through 2.0-mm screen and divided into two parts: one was stored at − 80 °C for molecular analysis, and the other used for determining soil chemical properties after air drying.

### Soil chemical properties and wheat yields

Soil pH was measured with a pH meter using a 1:1 sample:water extract, after being shaken for 2 h. Soil OM was determined according to the K_2_Cr_2_O_7_-capacitance method (Ciavatta et al. 1991). Total N (TN) was measured using the Kjeldahl method (Strickland and Sollins 1987). Nitrate N (NO_3_^−^-N) and ammonium N (NH_4_^+^-N) were quantified by extraction with 2 M KCl, steam distillation and titration (Swift et al. 1996). A modified resin-extraction method was used for AP analysis (Hedley and Stewart 1982). Available K (AK) was analyzed by flame photometry (Shen et al. 2013). Wheat yields were recorded by the Heilongjiang Academy of Agricultural Sciences, after harvest in September 2016.

### DNA extraction, PCR and Illumina MiSeq sequencing

A total of 12 soil samples were analyzed. A PowerSoil DNA extraction kit (MOBIO Laboratories Inc., Carlsbad, CA, USA) was used for DNA extraction from 0.25 g of soil of each sample according to the manufacturer’s method. Five successive extractions from each soil sample were combined together to obtain enough DNA and to minimize the DNA extraction bias. Purification was performed according to Zhou et al. (2016).

The AMF DNA was selectively amplified from each soil extractions by nested PCR (Williams et al. 2017). The first-round PCR with primers LR1 (5′-GCA TAT CAA TAA GCG GAG GA-3′) and FLR2 (5′-GTC GTT TAA AGC CAT TAC GTC-3′) was carried out in final 50-μL reaction mixtures containing 5 μL of 10 × PCR buffer, 4 μL of dNTPs (2.5 mM), 2 μL of each primer (10 μM), 0.75 U of Pyrobest DNA Polymerase and 30 ng of template DNA (Van Tuinen et al. 1998). The PCR protocol used was an initial denaturation at 95 °C for 5 min, 30 cycles of denaturation at 95 °C for 45 s, annealing at 58 °C for 50 s, extension at 72 °C for 45 s and a final extension at 72 °C for 10 min. The primers FLR3 (5′-TTG AAA GGG AAA CGA TTG AAG T-3′) and FLR4 (5′-TAC GTC AAC ATC CTT AAC GAA-3′) were used for the second-round PCR (Gollotte et al. 2004). The FLR3/FLR4 set contained a specific sequencing adapter followed by a 8 mer multiplexing identifier. The reaction mixtures and cycling conditions were the same as for the first round, with modifications of the template using the product of the first amplification. After purification, nested PCR products were quantified using QuantiFluor™-ST (Promega, USA) (Chen et al. 2016), and then normalized in equimolar ratios and sequenced using the Illumina MiSeq platform. Raw sequencing reads were deposited in the NCBI Sequence Read Archive with accession number SRX3209560.

### Bioinformatics and statistical analysis

We used Mothur 1.32 (http://www.mothur.org/) to process the sequencing data as described by Schloss et al. (2009). Barcode sequences were used to assign raw data by samples and low quality sequences were trimmed and excluded to obtain valid tags. Then, operational taxonomic units (OTUs) were defined by clustering at the 97% similarity level using UPARSE (Edgar 2013). Taxonomy was assigned to fungal OTUs according to the NCBI nucleotide sequences database. According to Camenzind et al. (2014) and Williams et al. (2017), the sequences out of *Glomeromycota*, as well as OTU singletons, were detected by manual BLASTing against the GenBank non-redundant nucleotide database and then removed. As a result, the sequences belonged to *Glomeromycota* phylum were obtained. The alpha diversity indices of AMF were calculated using Mothur (Schloss et al. 2009). A principal coordinate analysis (PCoA) on the basis of the weighted Fast UniFrac metric was carried out to examine the effect of inorganic and organic fertilizers on AMF community composition and to compare between-sample variations (Marsh et al. 2013). Redundancy analysis (RDA) was performed to visualize the effect of edaphic factors on AMF community structure using CANOCO 5.0 software.

SPSS (V19, Chicago, IL, USA) was used for statistical analysis. The differences in soil chemical properties, alpha diversity indices (Chao1, PD whole tree, Shannon) and AMF community abundances among samples were analyzed using one-way analysis of variance. Tukey’s procedure was followed for paired comparisons of different treatments. In all tests, *P* < 0.05 was considered significant.

## Results

### Soil chemical properties in wheat rhizosphere and wheat yields

Soil chemical properties in the wheat rhizosphere respond differently to fertilization regimes (Table 1). The AP concentrations were higher under the three fertilization treatments than that of CK, with the highest for NPK and MNPK treatments. The NPK treatment significantly increased AK and TN concentrations compared with CK. The NO_3_^−^-N concentration was significantly higher under the MNPK compared with the NPK treatment. With regards to soil pH, no significant differences were detected between CK and M treatment. Although the application of inorganic N, P and K fertilizer in both NPK and MNPK treatments significantly decreased soil pH, manure significantly alleviated soil acidification. The application of manure also significantly increased soil OM compared with CK. In addition, wheat yields were significantly higher under the three fertilization treatments compared with CK, with NPK and MNPK the most effective.

**Table 1:** Soil properties and wheat yield in different fertilization regimes

Treatment	pH	AP, g kg^−1^	AK, g kg^−1^	TN, g kg^−1^	NH_4_^+^-N, mg kg^−1^	NO_3_^−^-N, mg kg^−1^	OM, g kg^−1^	Yield, kg ha^−1^
CK	6.81 ± 0.01 c	10.36 ± 2.73 a	127.22 ± 6.91 a	1.41 ± 0.03 a	15.87 ± 2.46 a	0.93 ± 0.34 ab	28.14 ± 0.28 a	1546.4 ± 96.4 a
M	6.84 ± 0.09 c	20.28 ± 5.45 b	143.74 ± 8.25 ab	1.42 ± 0.05 ab	13.08 ± 2.10 a	1.04 ± 0.20 ab	29.49 ± 0.35 b	2927.9 ± 437.9 b
NPK	6.07 ± 0.06 a	76.08 ± 3.94 c	148.40 ± 3.54 b	1.52 ± 0.05 b	15.70 ± 2.15 a	0.50 ± 0.05 a	29.25 ± 0.46 ab	3938.3 ± 238.3 c
MNPK	6.26 ± 0.06 b	79.83 ± 3.64 c	153.80 ± 10.22 b	1.44 ± 0.02 ab	13.97 ± 0.99 a	1.15 ± 0.22 b	28.48 ± 0.80 ab	4104.9 ± 214.9 c

Values are means ± standard deviations (n = 3). Values within the same column followed by different letters indicate significant differences (*P* < 0.05) according to Tukey’s multiple comparison

Treatment: CK, no fertilizer; M, manure; NPK, chemical nitrogen, phosphorus and potassium fertilizer; MNPK, chemical nitrogen, phosphorus and potassium fertilizer plus manure

Soil properties: AP, available P; AK, available K; TN, total nitrogen; NH_4_^+^, ammonium nitrogen; NO_3_^−^-N, nitrate nitrogen; OM, organic matter

### Alpha diversity of AMF

A total of 381,228 raw tags were obtained from the Illumina MiSeq platform sequence of 12 soil samples. After removal of reads with a low quality score (< 20), ambiguous nucleotides, incomplete barcodes and chimeras, there were 355,011 high quality sequences with a range of read length of 320–360 bp produced, accounting for 93.1% of total sequences. All the high quality sequences were classified into 99 OTUs (97% similarity cutoff). Numbers of OTUs, Good’s coverage, richness and diversity under the different fertilization regimes are shown in Table 2. The Good’s coverage values exceeded 99.9%, and the numbers of OTUs under different fertilization regimes were in the range of 62.7–89.0. Moreover, the NPK treatment significantly decreased the richness index (Chao1). The application of manure led to the greatest values of Shannon diversity index, which was lowest for NPK. Compared with CK, the NPK treatment significantly decreased PD whole tree index.

**Table 2:** Numbers of observed operational taxonomic units, coverage, richness and diversity of soil in different fertilization regimes

Treatment	Goods coverage (%)	Operational taxonomic units	Richness	Diversity	
			Chao1	PD whole tree	Shannon
CK	99.93 ± 0.02 ab	89.0 ± 1.0 c	104.17 ± 4.56 b	6.37 ± 0.04 b	3.70 ± 0.23 ab
M	99.91 ± 0.01 a	78.3 ± 4.7 b	98.20 ± 5.14 b	5.81 ± 0.54 ab	3.97 ± 0.20 b
NPK	99.94 ± 0.01 b	62.7 ± 5.0 a	80.19 ± 12.43 a	5.00 ± 0.62 a	3.53 ± 0.18 a
MNPK	99.91 ± 0.02 a	71.7 ± 5.0 ab	104.82 ± 8.85 b	5.66 ± 0.26 ab	3.81 ± 0.26 ab

Values within the same column followed by different letters indicate significant differences (*P* < 0.05) according to Tukey’s multiple comparison

### Community composition at different taxonomic levels of AMF

In addition to altering alpha diversity of AMF, chronic fertilization affected their community composition as well. At the order or higher taxonomic levels, AMF community compositions were very similar under the different fertilization regimes. All members of AMF were affiliated with the phylum *Glomeromycota*, and more than 82% belonged to class *Glomeromycetes*, accounting for 82.4–91.8% of total sequences. Four different orders were detected: *Glomerales*, *Diversisporales*, *Archaeosporales* and *Paraglomerales*.

**Fig. 1:**
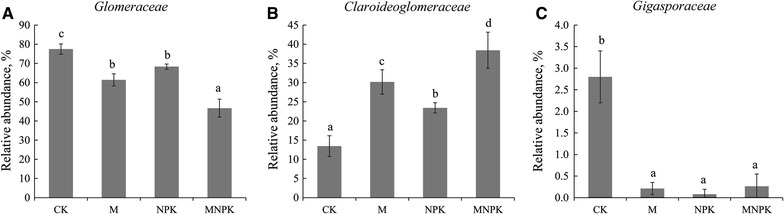
Relative abundances of AMF at family level with significant differences among fertilization regimes. **A** *Glomeraceae*; **B** *Claroideoglomeraceae*; **C** *Gigasporaceae*. Error bars indicate the standard deviation of relative abundance between three replicate samples. Lowercases above columns indicate significant differences (P < 0.05, Tukey’s test)

At the family level, the *Glomeraceae* were dominant, followed by *Claroideoglomeraceae*, *Gigasporaceae*, *Paraglomeraceae* and *Archaeosporaceae*. The families with significant differences among fertilization regimes are presented in Fig. 1. Abundances of *Glomeraceae* and *Gigasporaceae* were lower under all fertilization regimes compared with CK, but *Claroideoglomeraceae* exhibited the opposite pattern. Furthermore, both the M and MNPK treatments significantly increased the *Claroideoglomeraceae* abundance, with the highest for MNPK.

The AMF community compositions at genus level in response to chronic fertilization were quite different (Table 3, at least one group with a relative abundance > 1%). Compared with CK, the NPK treatment significantly increased the abundance of *Glomus*, but this decreased for MNPK. The abundances of *Funneliformis* were lower under all fertilization regimes compared with CK, with the lowest for NPK. Both inorganic fertilizer and manure significantly increased the abundance of *Rhizophagus*. The NPK treatment significantly decreased the abundance of *Septoglomus*, but manure increased it. It should be noted that the application of manure in both M and MNPK treatments had a significant positive effect on *Claroideoglomus* compared with CK and NPK treatments. In addition, there was a significant decline in *Gigaspora* abundance for all fertilization regimes compared with CK.

**Table 3:** Relative abundance of phylogenetic genera under different fertilization regimes

Class	Order	Family	Genus	CK (%)	M (%)	NPK (%)	MNPK (%)
*Glomeromycetes*	*Glomerales*	*Glomeraceae*	*Glomus*	31.56 ± 1.76 b	27.17 ± 3.22 ab	52.13 ± 2.66 c	22.85 ± 2.83 a
			*Funneliformis*	37.29 ± 2.72 d	17.35 ± 2.13 c	2.65 ± 1.23 a	10.77 ± 0.95 b
			*Rhizophagus*	6.94 ± 0.93 a	14.21 ± 0.97 c	12.50 ± 1.43 bc	11.26 ± 1.13 b
			*Septoglomus*	1.70 ± 0.22 b	2.80 ± 0.62 c	0.77 ± 0.20 a	1.79 ± 0.29 b
		*Claroideoglomeraceae*	*Claroideoglomus*	13.10 ± 0.97 a	30.17 ± 2.65 c	23.07 ± 2.46 b	38.09 ± 1.55 d
	*Diversisporales*	*Gigasporaceae*	*Gigaspora*	3.13 ± 1.10 b	0.21 ± 0.14 a	0.05 ± 0.06 a	0.26 ± 0.29 a

Values within the same row followed by different letters indicate significant differences (*P* < 0.05) according to Tukey’s multiple comparison

At least one group’s relative abundance is more than 1% of the total sequences

Treatment: CK, no fertilizer; M, manure; NPK, chemical nitrogen, phosphorus and potassium fertilizer; MNPK, chemical nitrogen, phosphorus and potassium fertilizer plus manure

### Beta diversity of AMF

**Fig. 2:**
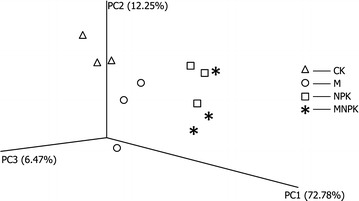
PCoA of the pyrosequencing reads based on the weighted Fast UniFrac metric. The first three axes are drawn and the percent of variance explained by each axis is given. Treatment: (triangle) CK, no fertilizer; (circle) M, manure; (square) NPK, inorganic phosphorus, potassium and nitrogen fertilizer; (star) MNPK, inorganic P, K and N fertilizer plus manure

The PCoA showed a clear separation among different fertilization regimes (Fig. 2). The axes of PC1, PC2 and PC3 explained 72.78, 12.25, and 6.47% of the variation, respectively. The plots of all fertilization regimes were well distributed along the PC1 axis with an order of CK, M, NPK and MNPK, indicating obvious shifts in AMF community composition. Moreover, AMF community compositions between treatments with or without inorganic fertilizer were quite different, suggesting a strong effect of inorganic inputs.

### Relationships between AMF community and edaphic factors

**Fig. 3:**
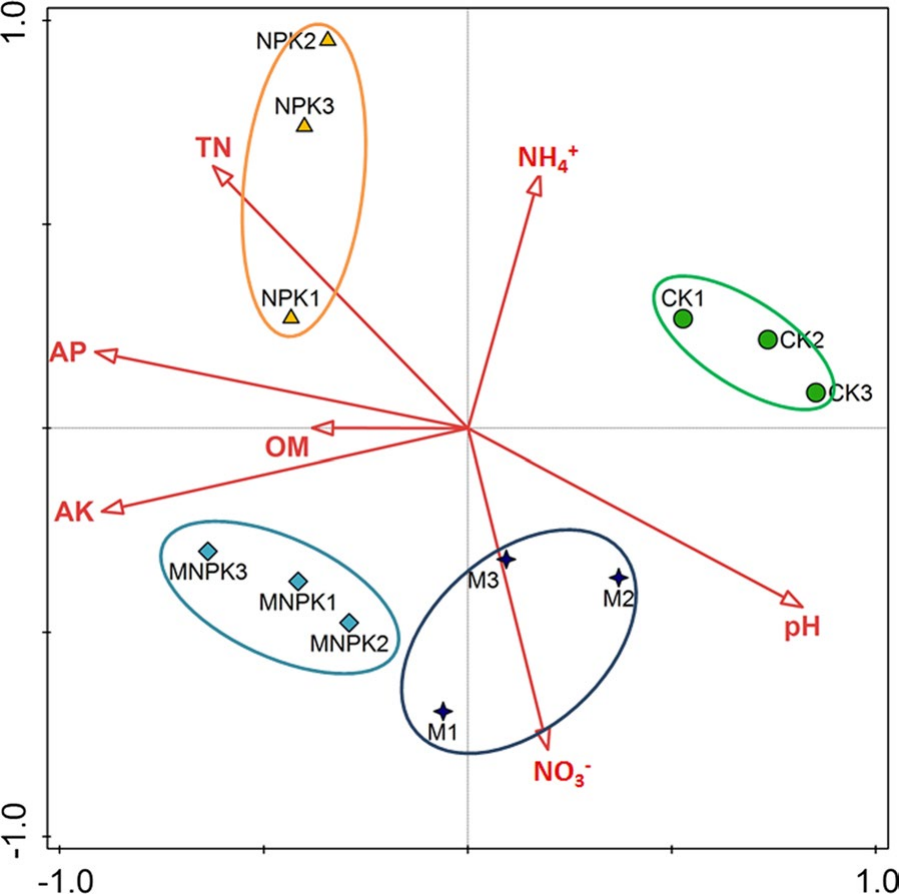
Redundancy analysis of soil AMF communities and soil properties. Soil factors indicated in red text include available phosphorus (AP), available potassium (AK), pH, soil concentration of NH_4_^+^ (NH_4_^+^), soil concentration of NO_3_^−^ (NO_3_^−^), total nitrogen (TN) and organic matter (OM). Treatment: (triangle) CK, no fertilizer; (circle) M, manure; (square) NPK, inorganic phosphorus, potassium and nitrogen fertilizer; (cross) MNPK, inorganic P, K and N fertilizer plus manure

The results of the RDA visualized the effects of edaphic factors on AMF community composition (Fig. 3). All the soil chemical properties selected in the current study accounted for 90.2% of the variation between samples. The first constrained axis explained 65.85% and the second explained 13.56% of the total variance. According to the forward-selection option in CANOCO with the Monte Carlo test, the most important contributors affecting the AMF community were soil AP (F = 11.1, *P* = 0.002), OM (F = 5.1, *P* = 0.012) and pH (F = 3.6, *P* = 0.026), accounting for 58.2, 19.0 and 10.5% of the variation, respectively. The other soil properties affected AMF community composition in the following order: NH_4_^+^-N > NO_3_^−^-N > TN > AK.

## Discussion

### Chronic fertilization changed rhizosphere soil pH and OM

Soil properties in the wheat rhizosphere differed significantly among fertilization regimes. Soil pH played a key role in soil quality and crop production. Although soil pH was significantly higher for the MNPK than the NPK treatment, both treatments significantly decreased soil pH compared with CK. This confirmed the positive impact of inorganic fertilizer on soil acidification (Guo et al. 2010), as well as the positive effect of manure on alleviation of soil acidification, likely due to the buffering functions of organic acids, carbonates and bicarbonates (Whalen et al. 2000; Garcia-Gil et al. 2004). Furthermore, manure containing macronutrients obviously led to accumulation of soil OM (Xie et al. 2014).

### Chronic fertilization affected AMF richness and diversity

In the current study, chronic fertilization shifted alpha diversity of AMF, probable due to the changes of soil chemical properties. Inorganic P and N fertilizers supplied these soil resources for plants and so, in turn, fewer nutrients would be allocated to underground mycorrhizae (Johnson et al. 2013; Williams et al. 2017), leading to low AMF richness and diversity under NPK regium. Under enriched soil N and P conditions, AMF were found to reduce the numbers of arbuscules and extraradical hyphae (Johnson et al. 2008), and plants invested less in symbiotic development (Smith and Read 2010). This was probably due to less carbohydrates in root exudates when plants experienced high P levels (Bhadalung et al. 2005). Moreover, high N and K concentrations also promote negative effects of P on AMF (Guttay and Dandurand 1989). However, contrasting results of positive effects of N fertilization were reported by Zheng et al. (2014). This might be caused by the P level, because N fertilization was found to increase AMF species richness and diversity in P-limited soils, but caused reductions in P-rich soils (Egerton-Warburton et al. 2007; Cheng et al. 2013). In contrast, organic fertilization was beneficial for AMF richness (Verbruggen et al. 2010). As previously documented, OM additions tend to increase AMF colonization of plant roots and hyphal abundance in soils (Gosling et al. 2010), in turn, the hyphae preferentially proliferate in organic substrates in experimental microcosms (Hodge and Fitter 2010) and can capture N from organic substrates (Leigh et al. 2009). In addition, multiple lines of evidence suggest a key role for AMF in cycling nutrients via organic sources (Sheldrake et al. 2017).

The AMF functional traits are phylogenetically conserved (Powell et al. 2009), thus, the decreased alpha diversity of AMF probably result in loss or reduction in AMF biological functions (Williams et al. 2017). Additionally, the decline in AMF species diversity could increase ecosystem instability and P losses, and result in accumulation of fungal pathogens (Whipps 2004; Verbruggen et al. 2012), while decreasing the exchange of nutrients between plants and AMF (Maček et al. 2011) and so making soil more easily affected by disturbances related to climate change (De Vries et al. 2012). Plants may allocate biomass to the structures and stimulate the AMF symbiosis to acquire more nutrients given the limited resources or vice versa (Johnson et al. 2013). Consequently, the similar levels of available nutrients with NPK and MNPK treatments probably resulted in similar selection effects on AMF taxa, as well as no significant differences in diversity.

### Chronic fertilization shaped AMF community composition

Soil properties play a crucial role in shaping the rhizosphere AMF community (Qin et al. 2015). Selection by certain soil nutrients or competition between species can lead to changes in overall AMF abundance (Engelmoer et al. 2014) or even complete exclusion of particular species (Roger et al. 2013), resulting in shifts in AMF community composition. In our study, AMF community composition varied significantly among different fertilization regimes, in good agreement with previous findings (Verbruggen et al. 2010). The most common AMF family was *Glomeraceae*, which agreed well with other findings in arable fields and in several other temperate ecosystems (Verbruggen et al. 2010; Lin et al. 2012). *Claroideoglomeraceae* was positively related to manure application. The high abundance may have suppressed microbes with known pathogenic traits, as members within *Claroideoglomeraceae* (i.e., *Glomus etunicatum*) efficiently inhibited root disease caused by fungal pathogen (Sikes et al. 2009). Furthermore, *Claroideoglomeraceae* can also promote P uptake (Yang et al. 2015). In our previous study (Ding et al. 2017), various microbes besides *Glomeraceae* with known pathogenic traits also had lower levels following manure application. In comparison with CK, all fertilizer application significantly decreased the abundance of *Gigasporaceae*, which was negatively correlated with AP in soil (Verbruggen et al. 2010). This could be caused by plants allocating less biomass and energy to roots, thereby negatively affecting the AMF symbiosis under conditions of high AP in soil (Johnson et al. 2013).

At genus level, most taxa belonged to genus *Glomus* in our study, in accordance with previous findings (Manoharan et al. 2017), as *Glomus* can survive and propagate more easily due to the ability to colonize via pieces of mycelium or mycorrhizal root fragments (Daniell et al. 2001). Compared with other treatments, the abundance of *Glomus* was significantly higher in the NPK, which was similar to previous results (Liu et al. 2015b). Additionally, all fertilization treatments decreased *Funneliformis* abundance, while manure application showed significantly positive effect, probably due to the positive correlation with soil pH (Jansa et al. 2014). Manure application also benefited the abundance of *Claroideoglomus*, indicating positive effect on facilitating plants to assimilate more P in soil (Wang et al. 2017). The results confirmed other findings that *Claroideoglomus* was more abundant with organic than conventional farming as well (Manoharan et al. 2017). In addition, all fertilization treatments significantly increased *Rhizophagus* abundance, probably due to high levels of nutrients (Thonar et al. 2011). As Jansa et al. (2008) stated, species of *R. intraradices* within *Rhizophagus* showed the highest benefit to hosts, indicating a beneficial effect of fertilization, particularly using organic manure. In comparison with CK, all fertilizer treatments with a high level of AP showed lower abundance of *Gigaspora*. This is likely because sufficient soil nutrients, especially AP, could shift species composition in favor of less efficient mutualists by plants allocating less biomass and energy to roots (Johnson 1993).

### Relationships between soil properties and AMF community composition

AMF community compositions were strongly affected by fertilizer-driven changes in soil chemical properties. In turn, shifts in AMF composition could influence plant growth through nutrient turnover, disease incidence and disease suppression, as well as soil quality. For instance, AMF produce large amounts of insoluble glycoprotein, glomalin and polysaccharides, which contribute to aggregate stability (Xie et al. 2015). In the current study, the PCoA revealed a significant correlation between soil fertilization regime and the AMF community. The application of inorganic fertilizer, especially P and N inputs, strongly affected the AMF community (Wang et al. 2011; Chen et al. 2014). In comparison, less disturbance was observed under M treatment, due to the biological processes in the organically managed agriculture system (Harrier and Watson 2004). The results agreed well with previous findings that the AMF community was more similar to those occurring under organically fertilized fields than those under conventional management (Verbruggen et al. 2010).

The soil pH and AP were considered important contributors to shifts in the AMF community (Dumbrell et al. 2010; Xu et al. 2017). AP was crucial for AMF, as the P level in soil was directly associated with the allocation of nutrients and energy to underground mycorrhizae (Johnson et al. 2013; Williams et al. 2017). Meanwhile, the AMF played important roles in the P cycle. Most phosphate in soils is in the form of *ortho*-phosphate, which cannot be directly utilized by plants. This phosphate was absorbed by plant roots or by roots colonized by mycorrhizal fungi (Behie and Bidochka 2014). AMF can secrete enzymes that hydrolyze organic P compounds in soil. In addition, OM was also crucial in shaping the AMF community. As heterotrophs, AMF utilize exogenous C for growth. The soil OM and plant root exudates would have profound influences on AMF community structure. Soil pH was also an important factor that shaped the AMF community, which was in agreement with Xu et al. (2017).

## Abbreviations

AMF: arbuscular mycorrhizal fungi
N: nitrogen
P: phosphorus
K: potassium
C: carbon
CK: no fertilizer
M: manure
NPK: inorganic phosphorus, potassium and nitrogen fertilizer
MNPK: inorganic phosphorus, potassium and nitrogen fertilizer plus manure
OM: organic matter
TN: total nitrogen
AK: available potassium
AP: available phosphorus
NH_4_^+^-N: ammonium nitrogen
NO_3_^−^-N: nitrate nitrogen
OTUs: operational taxonomic units
PCoA: principal coordinates analysis
RDA: redundancy analysis

## References

[CR1] Bardgett R (2005). The biology of soil: a community and ecosystem approach.

[CR2] Behie SW, Bidochka MJ (2014). Nutrient transfer in plant-fungal symbioses. Trends Plant Sci.

[CR3] Bhadalung NN, Suwanarit A, Dell B, Nopamornbodi O, Thamchaipenet A, Rungchuang J (2005). Effects of long-term NP-fertilization on abundance and diversity of arbuscular mycorrhizal fungi under a maize cropping system. Plant Soil.

[CR4] Camenzind T, Hempel S, Homeier J, Horn S, Velescu A, Wilcke W, Rillig MC (2014). Nitrogen and phosphorus additions impact arbuscular mycorrhizal abundance and molecular diversity in a tropical montane forest. Glob Chang Biol.

[CR5] Chen Y, Zhang X, Ye J, Han H, Wan S, Chen B (2014). Six-year fertilization modifies the biodiversity of arbuscular mycorrhizal fungi in a temperate steppe in Inner Mongolia. Soil Biol Biochem.

[CR6] Chen Y, Zhao Z, Peng Y, Li J, Xiao L, Yang L (2016). Performance of a full-scale modified anaerobic/anoxic/oxic process: high-throughput sequence analysis of its microbial structures and their community functions. Bioresour Technol.

[CR7] Cheng W, Johnson DW, Fu S (2003). Rhizosphere effects on decomposition. Soil Sci Soc Am J.

[CR8] Cheng Y, Ishimoto K, Kuriyama Y, Osaki M, Ezawa T (2013). Ninety-year-, but not single, application of phosphorus fertilizer has a major impact on arbuscular mycorrhizal fungal communities. Plant Soil.

[CR9] Ciavatta C, Govi M, Antisari LV, Sequi P (1991). Determination of organic carbon in aqueous extracts of soils and fertilizers. Commun Soil Sci Plant Anal.

[CR10] Collins CD, Foster BL (2009). Community-level consequences of mycorrhizae depend on phosphorus availability. Ecology.

[CR11] Daniell TJ, Husband R, Fitter AH, Young JPW (2001). Molecular diversity of arbuscular mycorrhizal fungi colonising arable crops. FEMS Microbiol Ecol.

[CR12] De Vries FT, Liiri ME, Bjørnlund L, Bowker MA, Christensen S, Setälä HM, Bardgett RD (2012). Land use alters the resistance and resilience of soil food webs to drought. Nat Clim Chang.

[CR13] Ding J, Jiang X, Ma M, Zhou B, Guan D, Zhao B, Zhou J, Cao F, Li L, Li J (2016). Effect of 35 years inorganic fertilizer and manure amendment on structure of bacterial and archaeal communities in black soil of northeast China. Appl Soil Ecol.

[CR14] Ding J, Jiang X, Guan D, Zhao B, Ma M, Zhou B, Cao F, Yang X, Li L, Li J (2017). Influence of inorganic fertilizer and organic manure application on fungal communities in a long-term field experiment of Chinese Mollisols. Appl Soil Ecol.

[CR15] Dumbrell AJ, Nelson M, Helgason T, Dytham C, Fitter AH (2010). Relative roles of niche and neutral processes in structuring a soil microbial community. ISME J.

[CR16] Edgar RC (2013). UPARSE: highly accurate OTU sequences from microbial amplicon reads. Nat Methods.

[CR17] Egerton-Warburton LM, Johnson NC, Allen EB (2007). Mycorrhizal community dynamics following nitrogen fertilization: a cross-site test in five grasslands. Ecol Monogr.

[CR18] Engelmoer DJP, Behm JE, Toby Kiers E (2014). Intense competition between arbuscular mycorrhizal mutualists in an in vitro root microbiome negatively affects total fungal abundance. Mol Ecol.

[CR19] Garcia-Gil JC, Ceppi SB, Velasco MI, Polo A, Senesi N (2004). Long-term effects of amendment with municipal solid waste compost on the elemental and acidic functional group composition and pH-buffer capacity of soil humic acids. Geoderma.

[CR20] Gollotte A, van Tuinen D, Atkinson D (2004). Diversity of arbuscular mycorrhizal fungi colonising roots of the grass species *Agrostis capillaris* and *Lolium perenne* in a field experiment. Mycorrhiza.

[CR21] Gosling P, Ozaki A, Jones J, Turner M, Rayns F, Bending GD (2010). Organic management of tilled agricultural soils results in a rapid increase in colonisation potential and spore populations of arbuscular mycorrhizal fungi. Agric Ecosyst Environ.

[CR22] Guo JH, Liu XJ, Zhang Y, Shen JL, Han WX, Zhang WF, Christie P, Goulding KWT, Vitousek PM, Zhang FS (2010). Significant acidification in major Chinese croplands. Science.

[CR23] Guttay AJR, Dandurand LMC (1989). Interaction of the vesicular–arbuscular mycorrhizae of maize with extractable soil phosphorus levels and nitrogen–potassium fertilizers. Biol Fertil Soils.

[CR24] Hammesfahr U, Bierl R, Thiele-Bruhn S (2011). Combined effects of the antibiotic sulfadiazine and liquid manure on the soil microbial-community structure and functions. J Plant Nutr Soil Sci.

[CR25] Han X, Li H, Horwath WR (2013). Temporal variations in soil CO_2_ efflux under different land use types in the black soil zone of northeast China. Pedosphere.

[CR26] Harrier LA, Watson CA (2004). The potential role of arbuscular mycorrhizal (AM) fungi in the bioprotection of plants against soil-borne pathogens in organic and/or other sustainable farming systems. Pest Manag Sci.

[CR27] Hedley MJ, Stewart JWB (1982). Method to measure microbial phosphate in soils. Soil Biol Biochem.

[CR28] Hodge A, Fitter AH (2010). Substantial nitrogen acquisition by arbuscular mycorrhizal fungi from organic material has implications for N cycling. Proc Natl Acad Sci.

[CR29] Hodge A, Helgason T, Fitter AH (2010). Nutritional ecology of arbuscular mycorrhizal fungi. Fungal Ecol.

[CR30] Insam H, Gómez-Brandón M, Ascher J (2015). Manure-based biogas fermentation residues—friend or foe of soil fertility?. Soil Biol Biochem.

[CR31] Jansa J, Smith FA, Smith SE (2008). Are there benefits of simultaneous root colonization by different arbuscular mycorrhizal fungi?. New Phytol.

[CR32] Jansa J, Erb A, Oberholzer HR, Šmilauer P, Egli S (2014). Soil and geography are more important determinants of indigenous arbuscular mycorrhizal communities than management practices in Swiss agricultural soils. Mol Ecol.

[CR33] Johnson NC (1993). Can fertilization of soil select less mutualistic mycorrhizae?. Ecol Appl.

[CR34] Johnson NC, Wolf J, Koch GW (2003). Interactions among mycorrhizae, atmospheric CO_2_ and soil N impact plant community composition. Ecol Lett.

[CR35] Johnson NC, Rowland DL, Corkidi L, Allen EB (2008). Plant winners and losers during grassland *N*-eutrophication differ in biomass allocation and mycorrhizas. Ecology.

[CR36] Johnson NC, Angelard C, Sanders IR, Kiers ET (2013). Predicting community and ecosystem outcomes of mycorrhizal responses to global change. Ecol Lett.

[CR37] Kaschuk G, Kuyper TW, Leffelaar PA, Hungria M, Giller KE (2009). Are the rates of photosynthesis stimulated by the carbon sink strength of rhizobial and arbuscular mycorrhizal symbioses?. Soil Biol Biochem.

[CR38] Kim YC, Gao C, Zheng Y, He XH, Yang W, Chen L, Wan SQ, Guo LD (2015). Arbuscular mycorrhizal fungal community response to warming and nitrogen addition in a semiarid steppe ecosystem. Mycorrhiza.

[CR39] Leigh J, Hodge A, Fitter AH (2009). Arbuscular mycorrhizal fungi can transfer substantial amounts of nitrogen to their host plant from organic material. New Phytol.

[CR40] Li J, Mendoza A, Heine P (2008). Four-decade responses of soil trace elements to an aggrading old-field forest: B, Mn, Zn, Cu, and Fe. Ecology.

[CR41] Li T, Hu Y, Hao Z, Li H, Wang Y, Chen B (2013). First cloning and characterization of two functional aquaporin genes from an arbuscular mycorrhizal fungus *Glomus intraradices*. New Phytol.

[CR42] Lin X, Feng Y, Zhang H, Chen R, Wang J, Zhang J, Chu H (2012). Long-term balanced fertilization decreases arbuscular mycorrhizal fungal diversity in an arable soil in north China revealed by 454 pyrosequencing. Environ Sci Technol.

[CR43] Liu X, Burras CL, Kravchenko YS, Duran A, Huffman T, Morras H, Studdert G, Zhang X, Cruse RM, Yuan X (2012). Overview of Mollisols in the world: distribution, land use and management. Can J Soil Sci.

[CR44] Liu Y, Shi G, Mao L, Cheng G, Jiang S, Ma X, An L, Du G, Collins Johnson N, Feng H (2012). Direct and indirect influences of 8 yr of nitrogen and phosphorus fertilization on *Glomeromycota* in an alpine meadow ecosystem. New Phytol.

[CR45] Liu J, Sui Y, Yu Z, Shi Y, Chu H, Jin J, Liu X, Wang G (2015). Soil carbon content drives the biogeographical distribution of fungal communities in the black soil zone of northeast China. Soil Biol Biochem.

[CR46] Liu Y, Johnson NC, Mao L, Shi G, Jiang S, Ma X, Du G, An L, Feng H (2015). Phylogenetic structure of arbuscular mycorrhizal community shifts in response to increasing soil fertility. Soil Biol Biochem.

[CR47] Maček I, Dumbrell AJ, Nelson M, Fitter AH, Vodnik D, Helgason T (2011). Local adaptation to soil hypoxia determines the structure of an arbuscular mycorrhizal fungal community in roots from natural CO_2_ springs. Appl Environ Microbiol.

[CR48] Manoharan L, Rosenstock NP, Williams A, Hedlund K (2017). Agricultural management practices influence AMF diversity and community composition with cascading effects on plant productivity. Appl Soil Ecol.

[CR49] Marsh AJ, Sullivan OO, Hill C, Ross RP, Cotter PD (2013). Sequencing-based analysis of the bacterial and fungal composition of kefir grains and milks from multiple sources. PLoS ONE.

[CR50] Mueller RC, Bohannan BJM (2015). Shifts in the phylogenetic structure of arbuscular mycorrhizal fungi in response to experimental nitrogen and carbon dioxide additions. Oecologia.

[CR51] Powell JR, Parrent JL, Hart MM, Klironomos JN, Rillig MC, Maherali H (2009). Phylogenetic trait conservatism and the evolution of functional trade-offs in arbuscular mycorrhizal fungi. Proc R Soc Lond B Biol Sci.

[CR52] Qin H, Lu K, Strong PJ, Xu Q, Wu Q, Xu Z, Xu J, Wang H (2015). Long-term fertilizer application effects on the soil, root arbuscular mycorrhizal fungi and community composition in rotation agriculture. Appl Soil Ecol.

[CR53] Rillig MC (2004). Arbuscular mycorrhizae and terrestrial ecosystem processes. Ecol Lett.

[CR54] Rillig MC, Mummey DL (2006). Mycorrhizas and soil structure. New Phytol.

[CR55] Roger A, Colard A, Angelard C, Sanders IR (2013). Relatedness among arbuscular mycorrhizal fungi drives plant growth and intraspecific fungal coexistence. ISME J.

[CR56] Schloss PD, Westcott SL, Ryabin T, Hall JR, Hartmann M, Hollister EB, Lesniewski RA, Oakley BB, Parks DH, Robinson CJ (2009). Introducing mothur: open-source, platform-independent, community-supported software for describing and comparing microbial communities. Appl Environ Microbiol.

[CR57] Sheldrake M, Rosenstock NP, Revillini D, Olsson PA, Mangan S, Sayer EJ, Wallander H, Turner BL, Tanner EVJ (2017). Arbuscular mycorrhizal fungal community composition is altered by long-term litter removal but not litter addition in a lowland tropical forest. New Phytol.

[CR58] Shen Z, Zhong S, Wang Y, Wang B, Mei X, Li R, Ruan Y, Shen Q (2013). Induced soil microbial suppression of banana fusarium wilt disease using compost and biofertilizers to improve yield and quality. Eur J Soil Biol.

[CR59] Sikes BA, Cottenie K, Klironomos JN (2009). Plant and fungal identity determines pathogen protection of plant roots by arbuscular mycorrhizas. J Ecol.

[CR60] Singh H, Verma A, Ansari MW, Shukla A (2014). Physiological response of rice (*Oryza sativa* L.) genotypes to elevated nitrogen applied under field conditions. Plant Signal Behav.

[CR61] Smith SE, Read DJ (2010). Mycorrhizal symbiosis.

[CR62] Smith SE, Jakobsen I, Grønlund M, Smith FA (2011). Roles of arbuscular mycorrhizas in plant phosphorus nutrition: interactions between pathways of phosphorus uptake in arbuscular mycorrhizal roots have important implications for understanding and manipulating plant phosphorus acquisition. Plant Physiol.

[CR63] Strickland TC, Sollins P (1987). Improved method for separating light- and heavy-fraction organic material from soil. Soil Sci Soc Am J.

[CR64] Swift RS, Sparks DL (1996). Methods of soil analysis: part 3. Chemical methods. Soil Sci Soc Am B Ser.

[CR65] Thiele-Bruhn S, Bloem J, de Vries FT, Kalbitz K, Wagg C (2012). Linking soil biodiversity and agricultural soil management. Curr Opin Environ Sustain.

[CR66] Thonar C, Schnepf A, Frossard E, Roose T, Jansa J (2011). Traits related to differences in function among three arbuscular mycorrhizal fungi. Plant Soil.

[CR67] Treseder KK, Allen MF (2000). Mycorrhizal fungi have a potential role in soil carbon storage under elevated CO_2_ and nitrogen deposition. New Phytol.

[CR68] Van Der Heijden MGA, Bardgett RD, Van Straalen NM (2008). The unseen majority: soil microbes as drivers of plant diversity and productivity in terrestrial ecosystems. Ecol Lett.

[CR69] Van Tuinen D, Jacquot E, Zhao B, Gollotte A, Gianinazzi-Pearson V (1998). Characterization of root colonization profiles by a microcosm community of arbuscular mycorrhizal fungi using 25S rDNA-targeted nested PCR. Mol Ecol.

[CR70] Verbruggen E, Kiers ET (2010). Evolutionary ecology of mycorrhizal functional diversity in agricultural systems. Evol Appl.

[CR71] Verbruggen E, Röling WFM, Gamper HA, Kowalchuk GA, Verhoef HA, van der Heijden MGA (2010). Positive effects of organic farming on below-ground mutualists: large-scale comparison of mycorrhizal fungal communities in agricultural soils. New Phytol.

[CR72] Verbruggen E, Kiers ET, Bakelaar PNC, Röling WFM, van der Heijden MGA (2012). Provision of contrasting ecosystem services by soil communities from different agricultural fields. Plant Soil.

[CR73] Walder F, van der Heijden MGA (2015). Regulation of resource exchange in the arbuscular mycorrhizal symbiosis. Nat plants.

[CR74] Wang FY, Hu JL, Lin XG, Qin SW, Wang JH (2011). Arbuscular mycorrhizal fungal community structure and diversity in response to long-term fertilization: a field case from China. World J Microbiol Biotechnol.

[CR75] Wang X, Hoffland E, Feng G, Kuyper TW (2017). Phosphate uptake from phytate due to hyphae-mediated phytase activity by arbuscular mycorrhizal maize. Front Plant Sci.

[CR76] Whalen JK, Chang C, Clayton GW, Carefoot JP (2000). Cattle manure amendments can increase the pH of acid soils. Soil Sci Soc Am J.

[CR77] Whipps JM (2004). Prospects and limitations for mycorrhizas in biocontrol of root pathogens. Can J Bot.

[CR78] Williams A, Manoharan L, Rosenstock NP, Olsson PA, Hedlund K (2017). Long-term agricultural fertilization alters arbuscular mycorrhizal fungal community composition and barley (*Hordeum vulgare*) mycorrhizal carbon and phosphorus exchange. New Phytol.

[CR79] Wilson GWT, Rice CW, Rillig MC, Springer A, Hartnett DC (2009). Soil aggregation and carbon sequestration are tightly correlated with the abundance of arbuscular mycorrhizal fungi: results from long-term field experiments. Ecol Lett.

[CR80] Xie H, Li J, Zhu P, Peng C, Wang J, He H, Zhang X (2014). Long-term manure amendments enhance neutral sugar accumulation in bulk soil and particulate organic matter in a Mollisol. Soil Biol Biochem.

[CR81] Xie H, Li J, Zhang B, Wang L, Wang J, He H, Zhang X (2015). Long-term manure amendments reduced soil aggregate stability via redistribution of the glomalin-related soil protein in macroaggregates. Sci Rep.

[CR82] Xu X, Chen C, Zhang Z, Sun Z, Chen Y, Jiang J, Shen Z (2017). The influence of environmental factors on communities of arbuscular mycorrhizal fungi associated with *Chenopodium ambrosioides* revealed by MiSeq sequencing investigation. Sci Rep.

[CR83] Yang H, Zhang Q, Dai Y, Liu Q, Tang J, Bian X, Chen X (2015). Effects of arbuscular mycorrhizal fungi on plant growth depend on root system: a meta-analysis. Plant Soil.

[CR84] Zheng Y, Kim Y, Tian X, Chen L, Yang W, Gao C, Song M, Xu X, Guo L (2014). Differential responses of arbuscular mycorrhizal fungi to nitrogen addition in a near pristine Tibetan alpine meadow. FEMS Microbiol Ecol.

[CR85] Zhou J, Guan D, Zhou B, Zhao B, Ma M, Qin J, Jiang X, Chen S, Cao F, Shen D, Li J (2015). Influence of 34-years of fertilization on bacterial communities in an intensively cultivated black soil in northeast China. Soil Biol Biochem.

[CR86] Zhou J, Jiang X, Zhou B, Zhao B, Ma M, Guan D, Li J, Chen S, Cao F, Shen D, Qin J (2016). Thirty four years of nitrogen fertilization decreases fungal diversity and alters fungal community composition in black soil in northeast China. Soil Biol Biochem.

[CR87] Zhou J, Jiang X, Wei D, Zhao B, Ma M, Chen S (2017). Consistent effects of nitrogen fertilization on soil bacterial communities in black soils for two crop seasons in China. Sci Rep.

